# Patient safety and the value of pharmaceutical intervention in a cancer hospital

**DOI:** 10.1590/S1679-45082018AO4122

**Published:** 2018-04-19

**Authors:** Karina da Silva Aguiar, Jamile Machado dos Santos, Mônica Cristina Cambrussi, Solane Picolotto, Marcela Bechara Carneiro

**Affiliations:** 1 Hospital Erasto Gaertner, Curitiba, PR, Brazil.

**Keywords:** Economics, pharmaceutical, Pharmaceutical services, Medication errors, Drug prescriptions, Antineoplastic agents, Oncology service, hospital, Farmacoeconomia, Assistência farmacêutica, Erros de medicação, Prescrições de medicamentos, Antineoplásicos, Serviço hospitalar de oncologia

## Abstract

**Objective:**

To demonstrate economic impact of pharmaceutical evaluation in detection and prevention of errors in antineoplastic prescriptions.

**Methods:**

This was an observational and retrospective study performed in a cancer hospital. From July to August 2016 pharmacists checked prescriptions of antineoplastic and adjuvant drugs. Drug-related problems observed were classified and analyzed concerning drug, pharmaceutical intervention, acceptability and characteristic of the error. In case of problem related to dose, we calculated a deviation percentage related with correct dose and value spent or saved. Data were analyzed using descriptive statistics with frequency and percentage.

**Results:**

A total of 6,104 prescriptions and 12,128 medications were evaluated. Drug-related problems were identified in 274 (4.5%) prescriptions. Most of them was due to lack of information (n=117; 36.1%). Problems associated with dose accounted for 32.1% (n=98) of the total. In 13 cases (13.3%) ranging of prescribed dose was 50% greater than the correct dose. Intercepted drug-related problems provided savings of R$54.081,01 and expenses of R$20.863,36, therefore resulting in a positive balance of R$33.217,65. Each intervention promoted saving of R$126,78 with an acceptance rate of 98%. Main pharmaceutical interventions were information inclusion (n=117; 36.1%) and dose change (n=97; 29.9%). All errors were classified as error with no harm.

**Conclusion:**

Simple actions such as prescription checking are able to identify and prevent drug-related problems, avoid financial losses and add immeasurable value to patient safety.

## RESUMO

**Objetivo:**

Demonstrar o impacto econômico da avaliação farmacêutica na detecção e na prevenção de erros em prescrições de antineoplásicos.

**Métodos:**

Estudo observacional e retrospectivo realizado em um hospital oncológico. De julho a agosto de 2016, prescrições contendo antineoplásicos e fármacos adjuvantes ao tratamento foram avaliadas por farmacêuticos. Os problemas detectados relacionados a medicamentos foram classificados e analisados quanto ao medicamento, à intervenção farmacêutica, à aceitabilidade e à caracterização do erro. Quando o problema envolveu dose, calcularam-se a percentagem de desvio em relação à dose correta e o valor gasto ou economizado. Os dados foram analisados por estatística descritiva com aplicação de frequência e percentual.

**Resultados:**

Foram avaliadas 6.104 prescrições e 12.128 medicamentos. Identificaram-se problemas relacionados a medicamentos em 274 (4,5%) prescrições, sendo a maioria causado por falta de informações (n=117; 36,1%). Quando reunidos, os problemas envolvendo dose representaram 32,1% (n=98) do total. Em 13 casos (13,3%), a variação da dose prescrita em relação à correta foi maior do que 50%. Os problemas relacionados a medicamentos interceptados representaram economia de R$54.081,01 e gastos de R$20.863,36, resultando em saldo positivo de R$33.217,65. Cada intervenção promoveu economia de R$126,78 com aceitabilidade de 98%. As principais intervenções foram inclusão de informações (n=117; 36,1%) e alteração de dose (n=97; 29,9%). Todos os erros foram considerados sem dano.

**Conclusão:**

Ações simples de serem implantadas, como análise de prescrições, são capazes de identificar e prevenir problemas relacionados a medicamentos, evitar perdas financeiras e agregar imensurável valor na segurança do paciente.

## INTRODUCTION

The Brazilian Federal Pharmacy Council (CFF) defines pharmaceutical care as a set of pharmacist's actions in which the patient is the primary beneficiary.^(^ ^1^ ^)^ For this reason, pharmacists act more effectively in patient care and they are responsible, along with the health care multidisciplinary team, for safety and effectiveness of pharmacotherapy. This safety and effectiveness occurs by identification, resolution and prevention of drug-related problems (DRP).^(^ ^2^ ^)^

Drug-related problems can occur due to drug adverse reaction or medication errors (ME).^(^ ^3^ ^)^ Medication errors are avoidable events that might or might not cause harm to the patient, increase length of hospital stay and hospital-related costs.^(^ ^3^ ^,^ ^4^ ^)^

The Institute for Safe Medication Practices (ISMP) establishes that ME can be related with: lack of information about patient and his/her medication, miscommunication, errors in label, package, and name of medications, inadequate dispensation, storage, standardization, acquisition and use of medications, problems with administration devices, environmental factors, professionals' education and competence, patients' education, risk management and quality process.^(^ ^5^ ^)^

Among MEs, the prescription error is the one with high potential to cause harm to patients. Annually, about 44,000 to 98,000 Americans die because of MEs and, among these errors, from 2% to 14% affect hospitalized patients.^(^ ^6^ ^)^ The risk increases when prescription is incomplete. Therefore, to prevent ME and increase patient safety, is crucial adequate prescription.^(^ ^7^ ^)^

Pharmaceutical intervention (PI) with continuous pharmacotherapy monitoring can reduce DRP, increase effectiveness and decrease pharmacotherapy risks.^(^ ^2^ ^)^ Such practice is regulated by the CFF that defines as one of solely responsibility of pharmacists to evaluate medical prescription concerning amount, quality, compatibility, stability and interactions.^(^ ^8^ ^)^

To hospital, the ME represents important costs. For this reason, to identify its nature and causes is relevant to establish actions for ME prevention, particularly for potentially dangerous drugs.^(^ ^9^ ^)^

Pharmacoeconomics is an analytical tool often used for medication management. This tool is used to study economic factors influencing medication use also considering clinical endpoints.^(^ ^10^ ^)^

There are four types of economic analyses: (a) cost minimization in which are compared interventions with equivalent effectiveness, *i.e.*, differentiation only in costs, (b) cost-benefit in which both costs and benefits in health are measured in monetary units, and results are expressed as net profit, (c) cost-effectiveness in which interventions effects are compared with health results and costs, *i.e.*, a measurement between cost units and clinical benefits, (d) cost-utility in which measurement unit of clinical benefit comprises in a combined measurement of benefits in terms of time and quality of life. Examples of utility measures are: quality-adjusted life year or disability-adjusted life year.^(^ ^11^ ^)^

Another economic concept existing is named cost-opportunity. This concept is based on the principle that existing resources are limited or scarce. Therefore, resources used in a productive process will no longer be available in another chance of production. Cost-opportunity emphasizes the importance of avoid wastes and inadequate investments of health resources.^(^ ^12^ ^)^

Irational use of medicines is an important public health problem and pharmacists are professionals with potentiality to improve the use of medicines, and reduce morbidity and mortality and pharmacotherapy-related costs.^(^ ^12^ ^)^

Currently more than a hundred of cancer drugs are used. These drugs differ in chemical composition, targetcells, purpose for specific cancer types and side effects. Because of the high complexity involved in cancer treatment, the patient requires an interdisciplinary approach including integral care and guarantee of an efficient and safe treatment. Pharmacists' action is an important part of patient care mainly to prevent ME by checking medical prescription. This action also contributes with saving of resources associated with a more rational pharmacotherapy, and improvement of health promotion.^(^ ^13^ ^,^ ^14^ ^)^

## OBJECTIVE

To demonstrate economic impact of pharmaceutical evaluation in detection and prevention of errors in antineoplastic prescriptions.

## METHODS

**Figure 1:**
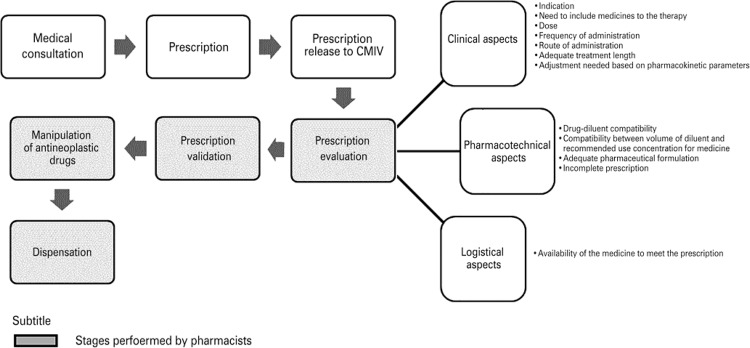
Flowchart of prescription evaluation

An observational and retrospective study was carried out in a teaching cancer specialized hospital with 120 beds located in South Brazil. Data were collected from July to August 2016. Sample included electronic prescription of antineoplastic and adjuvant drugs for cancer treatment prepared by the center for compounding intravenous medicines. The formula included mesna, calcium folinate, filgrastim, granisetron, and zoledronic acid. Prescriptions were received electronically by the pharmacists and subsequently checked and validated; the process is shown in the figure 1.

We included prescriptions of both adults and children hospitalized and outpatients with oncologic and hematological diseases.

Prescription were evaluated concerning the following parameters: (1) clinical, *i.e.*, indication, need of include a medication in the therapy, dose, frequency, route of administration, length of treatment and adjustment needed based on pharmacokinetic parameters, (2) pharmacotechnical parameters, *i.e.*, compatibility between diluent and drug, compatibility between volume of diluent and recommended concentration for medicines, pharmaceutical formulation and incomplete prescription, and (3) logistic, *i.e.*, availability of medicines.

To evaluate prescription we used as sources books, drug labels and scientific reports indexed in LILACS and MEDLINE.

Drug-related problems were recorded by pharmacists in a Microsoft Excel version 2017 spreadsheet and discussed with prescribers. This spreadsheet was storage in hospital electronic system, and all records were done in the same document. A periodically backup of document was done according to institutional routine of department of information technology.

**Figure 2:**
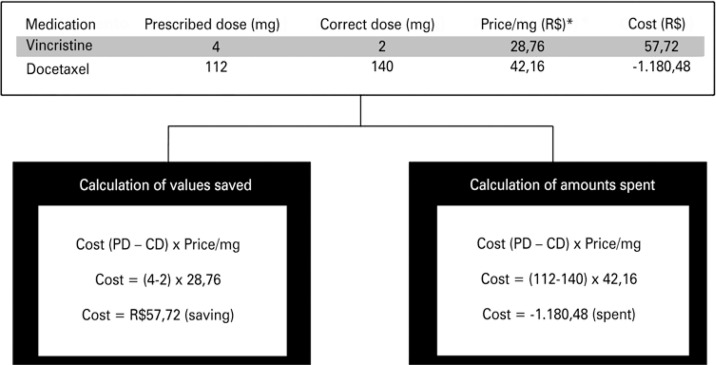
Method used to calculate costs

Collected variables were date and number of prescription, number of patient's medical record, type of care (hospitalizated or outpatient), drug used, DRP, PI, acceptability and characterization of error based on index by National Coordinating Council for Medication Error Reporting and Prevention (NCC MERP).^(^ ^4^ ^)^ In cases in which DRP involved dose we also registered the prescribed dose, the correct dose and percentage of deviation related with the correct dosage. In addition, total cost or value saved was calculated (Figure 2).

To calculate costs, we considered value of medicines and diluents, applied only to cases that PI resulted in change in dose and diluent, inclusion of medication in the prescription or medication suspension. The value used for such calculations was based on *Brasíndice*, version 861. We did not considered values of materials used in preparation of single dosages (needles, syringes, and infusion set).

The classification of DRP and PI was performed using a form adapted by the authors, based on the Pharmaceutical Care Network Europe (PCNE) Classification, version 6.2, in order to adequate them to DRP and PI characteristics identified in analyses of chemotherapy prescriptions protocols.^(^ ^15^ ^)^

Data were analyzed using descriptive statistics with application of frequency and percentage. Test of proportion was used for comparative analysis between prescriptions with errors and total of prescriptions.

## RESULTS

During the study, we evaluated 6,014 prescriptions. Of these, 274 (4.5%) had some type of ME (p<0.0001). In these prescriptions, there are 12,128 drugs and in 324 (2.7%) we identified ME. The main DRP identified are described in table 1.

**Table 1:** Main drug-related problems

Drug-related problems	n (%)
Incomplete prescription (*e.g.*: diluent and infusion time)	117 (36.1)
Subdose	35 (10.8)
Pharmacokinetic problem that required dose adjustment	34 (10.5)
Overdose	29 (9.0)
Incorrect infusion time	28 (8.6)
Duplicate prescription	27 (8.3)
Dose regimen with higher frequency than recommended	13 (4.0)
Incorrect volume of diluent	9 (2.8)
Inadequate route of administration	9 (2.8)
Dose regimen with lower frequency than recommended	5 (1.5)

A total of 44 different drugs had some type of DRP. The most common drugs are shown in table 2.

**Table 2:** Main medicines with drug-related problems

Drugs	n (%)
Zoledronic acid	47 (14.5)
Trastuzumab	43 (13.3)
Carboplatin	34 (10.5)
Cyclophosphamide	15 (4.6)
Doxorubicin	14 (4.3)
Fluorouracil	10 (3.1)
Calcium folinate	10 (3.1)
Gemcitabine	10 (3.1)
Methotrexate	10 (3.1)
MADIT-triple	10 (3.1)

MADIT-triple: intrathecal chemotherapy with dexamethasone, methotrexate and cytarabine.

The DRP involving prescribed dose, considering subdoses, overdoses, and non-adjusted doses according to pharmacokinetic problems resulted in identification of 98 DRP, and they represented 32.1% of the total.

Of 98 DRP involving dose, 49 (50%) had dose higher than the recommended. In 71 cases (72.4%), prescribed dose deviated in more than 10% of correct dose and, in 13 cases (13.3%), this raging was higher than 50%.

In terms of costs, we observed that DRP represented to institution a saving of R$54.081,01 and spending of R$20.863,36. The final positive balance was R$33.217,65. If such values were projected withing a 1-year-period, the saving would be of R$199.305,90.

To the scenario of intervention in which calculation of costs was considered, we observed that for these 262 medicines, each DRP along with PI, promoted a saving of R$126,78 to the institution.

The evaluation of items that caused higher reduction of expenses showed that about 70% of values saved were associated with interventions involving 5 medicines (trastuzumab, zoledronic acid, paclitaxel, rituximab and ifosfamide).

However, regard to increase in costs, we observed that 70% of costs were mainly related with filgrastim, paclitaxel, trastuzumab, carboplatin and docetaxel.

Acceptability of PI by medical team was 98%. The most performed interventions are shown in table 3.

**Table 3:** Main pharmaceutical interventions

Pharmaceutical interventions	n (%)
Inclusion of missing information	117 (36.1)
Dose change	97 (29.9)
Cancelation of prescription	43 (13.3)
Change in infusion time	25 (7.7)
Volume of diluent change	9 (2.8)
Change of route of administration	9 (2.8)
Initiation of new medicine	6 (1.9)
Change of frequency of administration	4 (1.2)
Medicine replacement	4 (1.2)
Diluent replacement	3 (0.9)

The NCC MERP states that errors can be categorized based on its capacity to cause harms to patients. Categories suggested are “no error”, “error but no harm”, “error with harm”, and “error with patient death”. In our study all errors were stopped before reach patients. For this reason, 100% of errors were classified as error with no harm.^(^ ^4^ ^)^

## DISCUSSION

Our study reaffirms the importance of pharmacists' contribution in actions for health promotion, protection and recovery, specially concerning analysis of prescriptions, PI along with health care team and activities to prevent DRP that could cause negative outcomes for patients' health.^(^ ^16^ ^,^ ^17^ ^)^

Our data showed a prescription error rate of 4.5% (p<0.0001). Such result agrees with those of 3.15% found by Ranchon et al.^(^ ^18^ ^)^ However, Mattsson et al., found lower values (1.6%) in electronic prescriptions.^(^ ^19^ ^)^ The highest value found in our study could be attributed to the fact that our study was carried out in a teaching hospital and also for features of the chemotherapy outpatient unit that, because of the high number of medical consultations, not always the physician who examined the patient is the same who writes the drug prescription. In such process, the lack of information and miscommunication favor errors.

The most relevant DRP in our study was caused by incomplete prescriptions, mainly lack of diluent and infusion time. A similar result was reported in the study by Silva in which most relevant errors were incomplete prescriptions, however, those more observed were related with dose and route of administration.^(^ ^20^ ^)^

Generally, drugs used in antineoplastic therapy have a narrow therapeutic index. The use of appropriate diluent and adequate infusion time are essential to achieve maximal therapeutic benefit and for its toxicity to remain within expected limits. Lack of such information in prescription can cause doubts and errors to other professionals such as pharmacists and nurses. For example, nursing team has the responsibility to check if doses dispensed by compounding intravenous medicines are corrected with the prescription. When information is not matching, the drug is not administered until double-checked with physician. This problem can cause a delay in administration or even loss of those medicines with low stability.

Another important aspect highlighted by a study include the 31 cases in which infusion time and volume of diluent were also incorrect, therefore, increasing risk of such errors to cause harm to patients.^(^ ^21^ ^)^

In our study we observed that DRP associated with zoledronic acid and carboplatin were found in 25% of identified errors. Such drugs have in common the fact that their doses are influenced by laboratorial tests of renal function in which could not be available at the time of prescription. The release of these results posterior to the date of medical prescription and non-evaluation of results by the prescriber were the main reason for occurrence of these DRP Similar results was found by Walsh et al. in a study that observed 90 errors, and zoledronic acid was seen in 21% of cases.^(^ ^22^ ^)^ In a study carried out by Ranchon et al., the carboplatin was the most related drug with ME, and it was associated to 21% of errors.^(^ ^18^ ^)^

Trastuzumab was also commonly found, however, PI was concentrated in prescribed diluent and infusion time in disagreement with manufactory recommendation and institutional protocol.

Cyclophosphamide, doxorubicin and calcium folinate were involved in DRP favored by parameterization of informatized system. In the institution where the study was carried out, prescriptions of antineoplastic and adjuvant drugs were parameterized for automatic inclusion of diluent and infusion times that are most commonly used in antineoplastic therapy protocols to adults in outpatient unit as well as to prioritize the most used measurement unit (*e.g.:* milligram). To pediatric prescriptions, in which occurred most of errors involving these medicines, there is a need of prescriber to change this information, a requirement that may favor errors.

Gemcitabine and methotrexate were more involved in errors related to incomplete prescription regarding infusion time, an event that could be severe for gemcitabine which could de more toxic when administered within a period higher than 60 minutes.^(^ ^23^ ^)^

Most of problems involving intrathecal administration occurred by omission of diluent in prescription of pediatric patients, as preconized by some protocols commonly used in pediatrics as well as standardized routine in our institution for this population. When all dosage errors were gathered, we observed that they represented 32.1% of total of errors, therefore, constituting the second most prevalent DRP. However, in a study done by Ranchon et al., 59.3% of errors were related to dose whereas in a reported by Vantard et al., this value was 54.1%.^(^ ^18^ ^,^ ^24^ ^)^

When percentage of deviation of prescribed doses were evaluated compared with correct prescription, we seen that many DRP could led to ineffective therapy as in 1.9% of cases that we suggested prescription of new medicine or in 15.4% in which the increase of the dose was suggested. Other extremely relevant situation would be morbidity potential or even the mortality of some detected DRP, if they were not identified and corrected. An example is: a prescribed dose of 9,820mg of ifosfamide when the correct dose would be 3,820mg; a prescribed dose of 4mg of vincristine when maximal allowed dose for this drug is 2mg; and 27 medicines that were prescribed in duplicity and they could be double administered, among other.

In such cases, the pharmacists' work avoided potential ME that might cause harm and these professionals also contributed extensively with safe use of medicines.

The main PI were related with omitted information, changes of dose and cancelation of prescription. Such results are similar to those presented by Delpeuch et al., in which main interventions were discontinuation of treatment (26.2%), dose adjustment (21.5%) and inclusion of medicines (16.9%).^(^ ^25^ ^)^

Our study identified a high acceptability of PI. In a study done by Nunes et al., that evaluated PI to prevent adverse events, the acceptability was 76%.^(^ ^26^ ^)^ In other two studies related with the subject, Néri et al., and Leape et al., reported 89% and 99% of acceptability, respectively. The high acceptability that occurred in our study can be attributed to the fact that the study was done in a teaching hospital and for the multidisciplinary team involved in patient care. Among these professionals was the pharmacist, therefore, reconfirming the importance role of this professional.^(^ ^27^ ^,^ ^28^ ^)^

In addition to DRP prevention, it is important to highlight that clinical service of prescriptions evaluation had a positive finance impact for the institution and this initiative was relevant to increase patient safety and accounted for savings in health resources. This type of saving is extremely important in current world scenario in which expenses with medicines are gradually increasing and every year even more health resources are required. In the United States, *e.g.*, the estimation of health-related expenses with medicines in 2015 were about US$457 billion, and this corresponded to 16.7% of expenses of general service with personal health.^(^ ^13^ ^)^

Our results, in terms of finance resources, corroborate with those identified in a study published in 2010 that investigated and described drug interaction, drug adverse reaction and DRP in an intensive care unit, and reported a saving estimated in R$510.000,00 within a 6 months period.^(^ ^29^ ^)^ In our study the estimated saving was R$33.217,65 within a 2 months period, considering that we evaluated only ME in antineoplastic and adjuvants prescriptions.

We also seen that this cost-related reduction was observed considering only costs related with medicines and diluents. If we evaluated the finance impact caused in terms of harm to patients, in case we have not stopped DRP, costs would be even greater. Ranchon et al., evaluated potential costs of ME identified in French health system and observed that the amount would be about 92 million Euros per year, and it would lead to 216 additional days of hospitalization.^(^ ^18^ ^)^

Our study was able to show that implement a pharmaceutical service of prescription evaluation in antineoplastic therapy service is crucial, and such service should be encouraged because in addition to improve patient safety it may promote important finance savings, and, therefore lead to a less costly antineoplastic chemotherapy for health system and a chance to enlarge the number of patients who can benefit of this treatment.

This study limitations are related with the single analysis considering direct costs with medicines and diluents.

Further studies can evaluate cost related with hospital care, payments for health team members, adverse events or changes in individuals productivity in case of non-intercepted ME. If such limitations were eliminated from our study, finance savings would be even higher. Drug-related problems were identified by evaluation of prescription without follow-up of the patient by clinical pharmacists. If a follow-up was done, the identification of DRP, adverse reactions, among others would be improved.

## CONCLUSION

Health is priceless. However, there is a need to recognize that costs exist and available resources should be better allocated. Simple actions such as pharmaceutical interventions and prescription evaluation can identify drug-related problems, prevent adverse events, reduce finance losses and add immeasurable value to patient safety. Patient safety is a dynamic target and approaches to achieve such goal must continue to evolve in order to improve even more pharmaceutical care delivered.
